# Accessing isotopically labeled proteins containing genetically encoded phosphoserine for NMR with optimized expression conditions

**DOI:** 10.1016/j.jbc.2022.102613

**Published:** 2022-10-17

**Authors:** Cat Hoang Vesely, Patrick N. Reardon, Zhen Yu, Elisar Barbar, Ryan A. Mehl, Richard B. Cooley

**Affiliations:** 1 GCE4All Research Center, Oregon State University, Corvallis, Oregon, USA; 2 Department of Biochemistry and Biophysics, Oregon State University, Corvallis, Oregon, USA; 3 Oregon State University NMR Facility, Oregon State University, Corvallis, Oregon, USA

**Keywords:** post-translation modification, genetic code expansion, protein phosphorylation, phosphoserine, nuclear magnetic resonance, protein synthesis, protein expression, AIM, auto-induction media, GCE, genetic code expansion, HSQC, heteronuclear single quantum coherence, IDR, intrinsically disordered region, MIM, manual induction media, NIM, non-inducing media, NOESY, nuclear overhauser effect spectroscopy, OP, optical density, pSer, phosphoserine, sfGFP, super folder GFP

## Abstract

Phosphoserine (pSer) sites are primarily located within disordered protein regions, making it difficult to experimentally ascertain their effects on protein structure and function. Therefore, the production of ^15^N- (and ^13^C)-labeled proteins with site-specifically encoded pSer for NMR studies is essential to uncover molecular mechanisms of protein regulation by phosphorylation. While genetic code expansion technologies for the translational installation of pSer in *Escherichia coli* are well established and offer a powerful strategy to produce site-specifically phosphorylated proteins, methodologies to adapt them to minimal or isotope-enriched media have not been described. This shortcoming exists because pSer genetic code expansion expression hosts require the genomic Δ*serB* mutation, which increases pSer bioavailability but also imposes serine auxotrophy, preventing growth in minimal media used for isotopic labeling of recombinant proteins. Here, by testing different media supplements, we restored normal BL21(DE3) Δ*serB* growth in labeling media but subsequently observed an increase of phosphatase activity and mis-incorporation not typically seen in standard rich media. After rounds of optimization and adaption of a high-density culture protocol, we were able to obtain ≥10 mg/L homogenously labeled, phosphorylated superfolder GFP. To demonstrate the utility of this method, we also produced the intrinsically disordered serine/arginine-rich region of the SARS-CoV-2 Nucleocapsid protein labeled with ^15^N and pSer at the key site S188 and observed the resulting peak shift due to phosphorylation by 2D and 3D heteronuclear single quantum correlation analyses. We propose this cost-effective methodology will pave the way for more routine access to pSer-enriched proteins for 2D and 3D NMR analyses.

Phosphorylation, the most common type of post-translation modification, is an essential protein regulatory mechanism in eukaryotic cells (1, 2, 3). Dysregulation of phosphorylation-dependent signaling systems is linked to numerous disease pathologies as these post-translational modifications play key roles in cellular processes such as protein synthesis, signal transduction, and cell development (4, 5, 6). More than two-thirds of proteins in the human proteome undergo reversible phosphorylation and of these proteins, nearly 80% were identified at serine residues (7). Most phosphoserine (pSer) sites are located within flexible or disordered regions of proteins (8, 9), where they serve as regulatory switches to modulate conformational dynamics, function, and allosteric interactions (10, 11, 12, 13). NMR spectroscopy is the ideal technique for probing dynamics and structure of intrinsically disordered regions (IDRs)/intrinsically disordered proteins at the molecular level (14). Despite the ubiquitous nature of phosphorylation, few NMR studies focus on understanding the molecular consequences of IDR/intrinsically disordered protein phosphorylation. This is, in large part, caused by a lack of standardized and routine methods for synthesizing isotopically labeled, site-specifically phosphorylated proteins for NMR characterization.

The study of phospho-proteins by NMR requires that they be isotopically enriched with ^15^N and/or ^13^C and be homogenously phosphorylated at the targeted site(s). Standard expression strains of *Escherichia coli* (*e.g.*, BL21(DE3)) can biosynthesize all 20 natural amino acids from fundamental carbon and nitrogen building blocks so that protein expression in fully-defined minimal media containing ^13^C-sugars and ^15^NH_4_Cl provides a convenient strategy to produce isotopically labeled proteins. Site-specific phosphorylation of target proteins, on the other hand, continues to pose a challenge because the required kinase(s) is not always known, they may lack required specificity and they may not be easily isolated in a functional state for *in vitro* utility (14, 15, 16). Phosphomimetic mutations (Ser or Thr to Asp/Glu) are commonly introduced at the site of phosphorylation to overcome this issue, however, Asp or Glu do not faithfully recapitulate the geometry nor the charge density (17, 18, 19) and so they commonly misinform on the functional effects of authentic phosphorylation (20, 21, 22, 23, 24, 25).

Genetic code expansion (GCE) has emerged as a leading technology for the production of phosphorylated proteins because it allows site-specific, homogenous, and efficient translational incorporation of phosphorylated amino acids into any protein (Fig. 1*A*) (26, 27, 28, 29). In 2015, a high-efficiency GCE system was developed to translationally install pSer in response to an amber (TAG) stop codon, yet to date, this system has not been adopted to produce isotopically labeled phosphorylated proteins (26). Reasons for this have not been articulated in the literature, but we hypothesized they were rooted in the fact that pSer GCE expression systems utilize serine auxotrophic strains for phospho-protein expression, which means they cannot grow in minimal media (26, 27, 29, 30). Serine auxotroph expression hosts are used because charged, phosphorylated amino acids like pSer do not traverse from the media into the cell effectively, resulting in low bioavailability of the free amino phospho-amino acid (26, 27, 29, 30, 31). By deleting the *serB* gene, which hydrolyzes pSer to produce serine as the last step of serine biosynthesis, pSer accumulates inside the cell, providing a sufficient pool of pSer to feed the GCE machinery (31) (Fig. 1*A*). Serine supplementation should overcome this issue of auxotrophy, though isotopically labeled serine is expensive and serine feedback inhibition of SerA shuts down production of pSer (Fig. 1*A*) (32, 33, 34). Intracellular levels of pSer in WT *E. coli* strains are remarkably low having an intact SerB (31), and even if pSer media supplementation could overcome this issue, isotopically labeled pSer is not commercially available to our knowledge.

**Figure 1:**
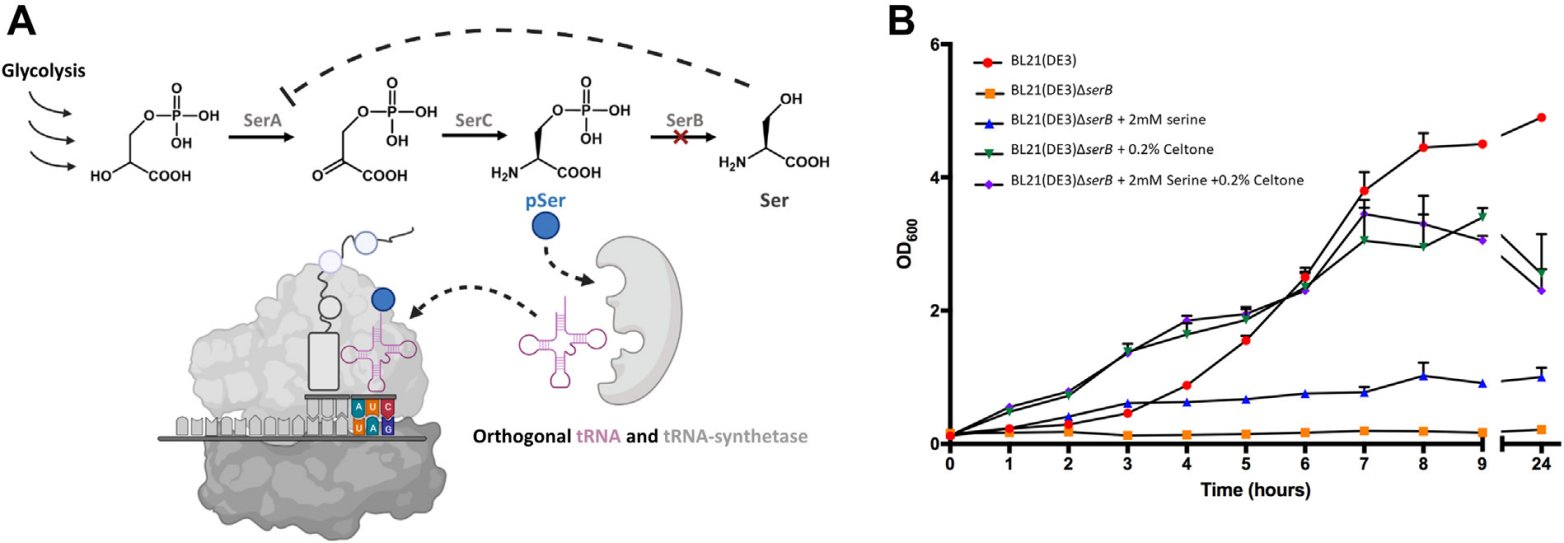
***Escherichia coli* Δ*serB* expression strains growth in minimal media with key supplements.***A*, deletion of *serB* allows phosphoserine concentrations to build up inside the cell for sufficient translational incorporation at programmed amber codon(s) with GCE-derived pSer machinery. However, serine feedback inhibition of SerA prevents build-up of pSer inside the cell. *B*, growth rate comparison of expression strains BL21(DE3) (*red*) and BL21(DE3) Δ*serB* (*orange*) with serine (*blue*), Celtone (*green*), or both (*purple*) over 24 h. Error bars represent SDs of cultures performed in duplicate. GCE, genetic code expansion; pSer, phosphoserine.

Needed therefore is an expression methodology that merges existing pSer GCE systems with methods for expressing isotopically labeled proteins. Here, we overcome these challenges to formulate an efficient and low-cost expression strategy that is sufficient for production of ^15^N-labeled proteins with site-specific pSer incorporated using serine auxotroph BL21(DE3) Δ*serB* as the expression host. This method can be easily adapted for ^13^C labeling and should accelerate access to phosphorylated proteins for NMR structural biology projects.

## Results

### Optimization of BL21(DE3) Δ*serB* growth in labeling media

We use here the *E. coli* expression host BL21(DE3) Δ*serB*, with the pSer phosphatase *serB* deleted to increase the intracellular levels of pSer (31) and thus, improve pSer incorporation into recombinant isotopically labeled protein (26, 28). Our first goal was to compare the growth rate between BL21(DE3) WT and BL21(DE3) Δ*serB* hosts in unlabeled minimal media without antibiotics and identify key supplements required for optimal BL21(DE3) Δ*serB* growth (Fig. 1*B*). We tracked optical density (OD) at 600 nm (OD_600_) over 24 h and confirmed WT BL21(DE3) cells grew robustly in the minimal media, while BL21(DE3) Δ*serB* would not grow unless supplemented with 2 mM serine, 0.2% (w/v) Celtone base powder, or both (Fig. 1*B*). Celtone base powder (referred to hereafter as Celtone) is an algal hydrolysate containing a mix of amino acids and used here because it is available with ^13^C and/or ^15^N enrichment. Interestingly, serine supplementation alone to BL21(DE3) Δ*serB* did not fully rescue growth phenotype to those of WT BL21(DE3), as indicated by an impaired growth rate and a low final cell density (final OD_600_ ∼1 *versus* ∼5, respectively). On the other hand, the addition of Celtone alone or in combination with serine improved growth rates and final cell densities that were comparable growth to BL21(DE3) WT. With these data, we reasoned that a minimal media supplemented with either both 2 mM serine and 0.2% (w/v) Celtone, or just Celtone alone, could be used for phospho-protein production with further expression optimization.

### Optimization of expression conditions for phosphorylated protein production

**Figure 2:**
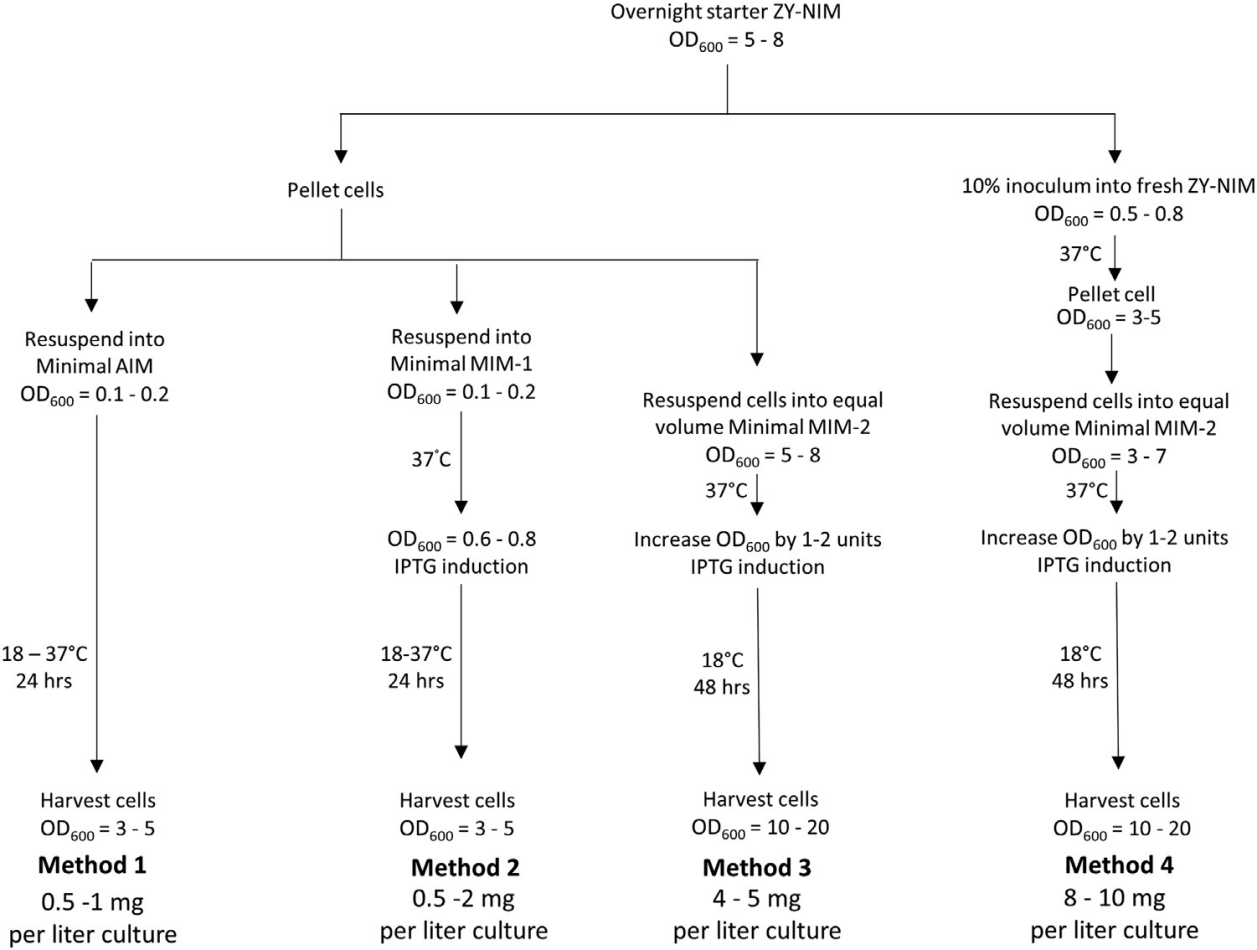
**Schematic of the four primary experimental expression strategies evaluated in this study**.

Having identified media for robust BL21(DE3) Δ*serB* growth, we set out to evaluate pSer protein expression using the high efficiency GCE system created by Chin *et al.* (26). This system utilizes the pKW2-EFSep plasmid which expresses the pSer tRNA synthetase variant SepRS-2, the amber codon suppression tRNA Sep-tRNA_CUA_(B4), and an EFTu variant enabling efficient delivery of pSer-aminoacylated tRNA to the ribosome, EF-Sep. To easily evaluate phospho-protein expression parameters, we expressed the fluorescent reporter super folder GFP (sfGFP) containing an amber TAG codon at position N150 (sfGFP-150TAG) from the pRBC plasmid (28). Previously, we found that rich auto-induction media (called ZY-AIM, Table 1) provided maximal levels of homogenously phosphorylated protein with this pSer GCE system (28), and so here, we first tested auto-induction expression strategies with Celtone- and serine-supplemented media amenable to isotopic labeling (Minimal AIM, Table 2, Method 1 in Fig. 2). In parallel, we also tested manual induction expression strategies in a minimal media similarly supplemented with Celtone and serine (MIM-1, Table 2, Method 2 in Fig. 2). For these expressions, starter cultures were grown overnight (∼16–18 h) in a rich non-inducing media (ZY-NIM, Table 1). Cells were then pelleted, resuspended in their respective expression minimal media. Protein expression yields were quantified by whole-cell fluorescence. The homogeneity of phosphorylation was assessed by Phos-tag gel electrophoresis of purified sfGFP proteins. With Phos-tag gels, phosphorylated proteins migrate slower than nonphosphorylated proteins permitting near-quantitative evaluation of protein phosphorylation status (35). For comparison, phosphorylated sfGFP was also expressed in rich ZY-AIM (28).

**Table 1:** Components for 50 mL of ZY non-inducing (ZY-NIM) and ZY auto-inducing media (ZY-AIM)

Component	For 50 ml of ZY-NIM	For 50 ml of ZY-AIM
ZY media^a^	45 ml	45 ml
1 M MgSO_4_^b^	0.1 ml	0.1 ml
25x M-Salts-1^c^	2.0 ml	2.0 ml
25 g/L NH_4_Cl^b^	2.0 ml	2.0 ml
40% (w/v) Glucose^d^	0.625 ml	-
50x 5052^e^	-	1.0 ml
5000x Trace Metals^f^	10 μl	10 μl

Components were sterilized separately and combined as follows after they are cooled to room temperature and immediately before use.

a ZY Media, per liter: 10 g Tryptone, 5 g Yeast Extract. Autoclave.

b Unlabeled. Sterile by autoclaving or filtering.

c 25x M-Salts-1: 0.625 M Na_2_HPO_4_, 0.625 M KH_2_PO_4_, 0.125 M Na_2_SO_4_. No pH adjustment. Autoclave.

d 40% (w/v) glucose: 20 g α-D-glucose in total volume of 50 mL. Autoclave or sterile filter.

e 50x 5052, per 500 mL: 12.5 g α-D-glucose, 50 g α -lactose, 125 mL glycerol (or 250 mL of 50% glycerol, which is easier to measure). Note: to get the lactose to dissolve, heat gently in microwave. Once dissolved, lactose will stay in solution indefinitely.

f Not essential for ZY-NIM, but can help cell growth, as previously described (28).

**Table 2:** Components for 50 mL of minimal auto-induction and minimal manual-induction media

Component	For 50 ml		
	Minimal AIM	Minimal MIM-1 (low density)	Minimal MIM-2 (high density)
Sterile Water	45 ml	45 ml	45 ml
1 M MgSO_4_	0.1 ml	0.1 ml	0.1 ml
25x M-Salts-1	2.0 ml	2.0 ml	-
25x M-Salts-2 (high pH)^a^	-	-	2.0 ml
25 g/L NH_4_Cl OR^15^NH_4_Cl	2.0 ml	2.0 ml	2.0 ml
40% (w/v) Glucose (Unlabeled OR ^13^C)	-	0.625 ml	1.25 ml
50x 5052	1.0 ml	-	-
10% (w/v) CELTONE (Unlabeled, ^15^N OR^15^N/^13^C)^b^	1.0 ml	1.0 ml	1.0 ml
5000x Trace Metals	10 μl	10 μl	10 μl

Components were sterilized separately and combined as follows after they cooled to room temperature and immediately before use.

a 25x M-Salts 2 (high pH): Same as 25x M-salts-1 (see Table 1), pH adjusted to 8 to 8.2 with 10 M KOH. Autoclave.

b Dissolve Celtone powder in sterile water to 10% (w/v) final concentration. Do not autoclave.

**Figure 3:**
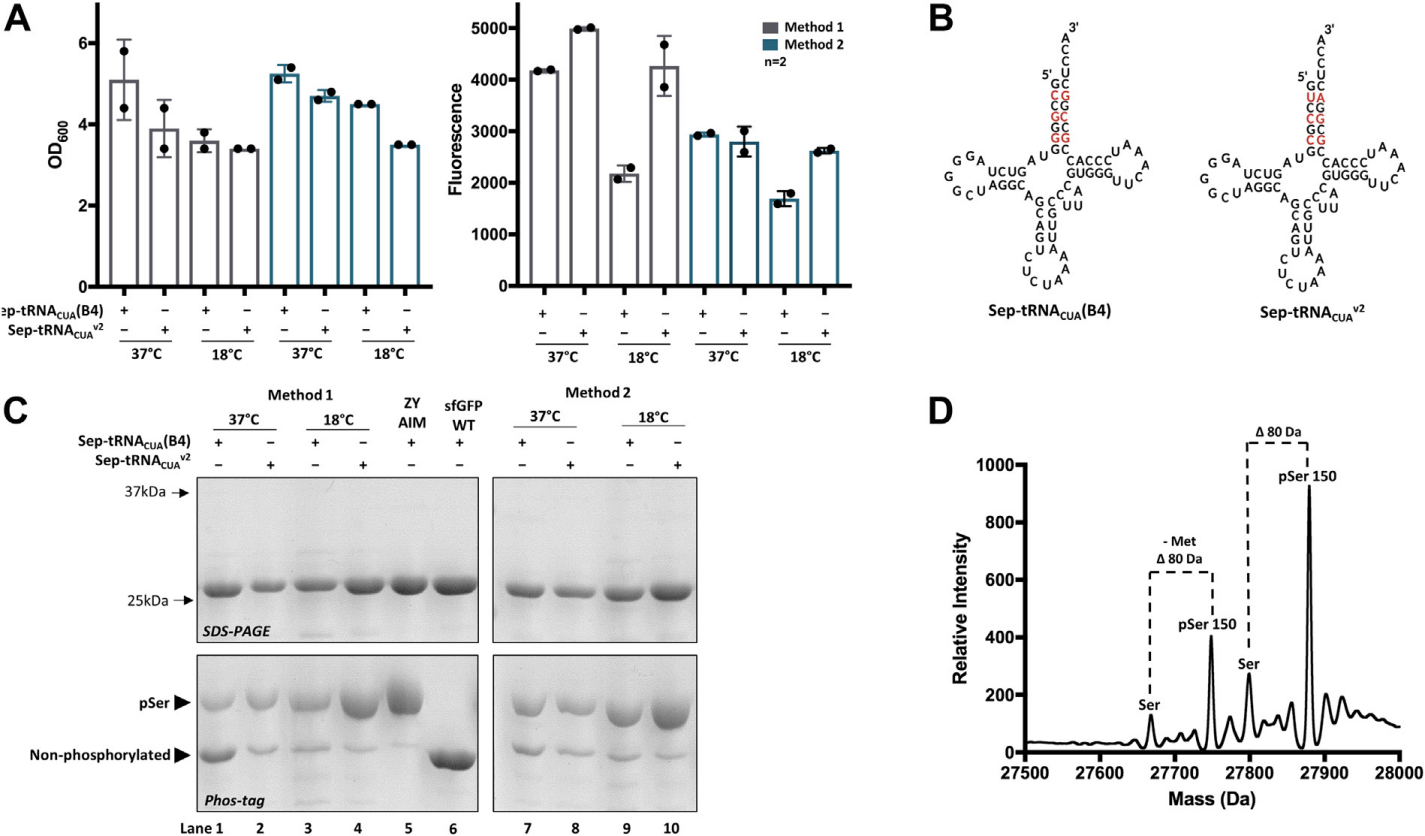
**Evaluation of phosphoserine incorporation into sfGFP in low density cultures (Methods 1 and 2).***A*, comparison of final cell growth density and sfGFP protein production in supplemented media expressed at 37 °C and 18 °C using Methods 1 (*gray bars*) and 2 (*navy bars*). Error bars represent SDs of expressions performed in duplicate. *B*, Sep-tRNA_CUA_(B4) and Sep-tRNA_CUA_^v2^ sequence and structure showing mutations in the acceptor stem previously shown to minimize mis-acylation by endogenous host tRNA-synthetases (27). *C*, 12% SDS-PAGE and Phos-tag gels of purified sfGFP proteins from panel *A* (for full gels, see Fig. S1). Slower electrophoretic mobility on Phos-tag gel indicates site-specific incorporation of pSer. Site-specific incorporation of pSer is confirmed with slower electrophoretic mobility on Phos-tag gel and *D*, whole-protein mass spectrometry. Measured and expected whole-protein masses of sfGFP-150pSer produced from method 2 at 37 °C (lane 8) are 27879.81 and 27882.00 Da, respectively. *Dashed lines* indicate 80 Da difference corresponding to the removal of a phosphate group from phosphatase activity. pSer, phosphoserine; sfGFP, super folder GFP.

All expression cultures grown at 37 °C grew to a similar OD_600_ of ∼4 to 5 and with total cell fluorescence about 30% higher in the auto-induction expression, corresponding to 1 to 2 mg of protein per liter culture (Fig. 3*A*). However, only ∼40% of purified sfGFP expressed from auto-induction and ∼60% from manual-induction were phosphorylated, in stark contrast to >95% of the protein being phosphorylated when expressed in and purified from rich ZY-AIM media (Fig. 3*C* lanes 1, 7 and 5 respectively). We assumed the source of nonphosphorylated protein could come from the following: (1) near-cognate suppression by endogenous tRNAs at encoded amber codons, (2) protein dephosphorylation from phosphatase activity, and (3) mis-aminoacylation of the Sep-tRNA_CUA_(B4) by endogenous aminoacyl-tRNA synthetases. Near cognate suppression of amber stop codons is generally insignificant when using expression hosts such as BL21(DE3) Δ*serB* that contain Release Factor 1 (RF1), the *E. coli* protein responsible for terminating translation at amber codons (28, 36). We next considered the possibility that the our GCE tRNA, Sep-tRNA_CUA_(B4), was mis-aminoacylated by endogenous synthetases in these conditions where overall incorporation efficiency was low (27). We addressed this by replacing Sep-tRNA_CUA_(B4) with the Sep-tRNA_CUA_^v2^ (Fig. 3*B*), a variant containing mutations in its acceptor stem that minimize mis-acylation by endogenous synthetases (27). Using this strategy, we re-expressed sfGFP-150TAG in the same auto- and manual-induction media at 37 °C. While expression yields were comparable with Sep-tRNA_CUA_^v2^, measurable improvements in incorporation fidelity were observed, with ∼80% of the purified sfGFP being phosphorylated (Fig. 3*C*, lanes 2 and 8). The remaining nonphosphorylated protein was identified by mass spectrometry to be exactly 80 Da lighter, consistent with hydrolysis of pSer by cellular phosphatases during expression (Fig. 3*D*). We found that by conducting the expressions at 18 °C instead of 37 °C, cellular phosphatase activity was notably decreased, with >90% of the purified sfGFP being phosphorylated using Methods 1 and 2 with the Sep-tRNA_CUA_v2 (Fig. 3*B*, lanes 4 and 10). Having improved homogeneity of phospho-protein expression, we next set out to optimize expression yield.

### Optimizing phospho-protein yield for NMR applications

**Figure 4:**
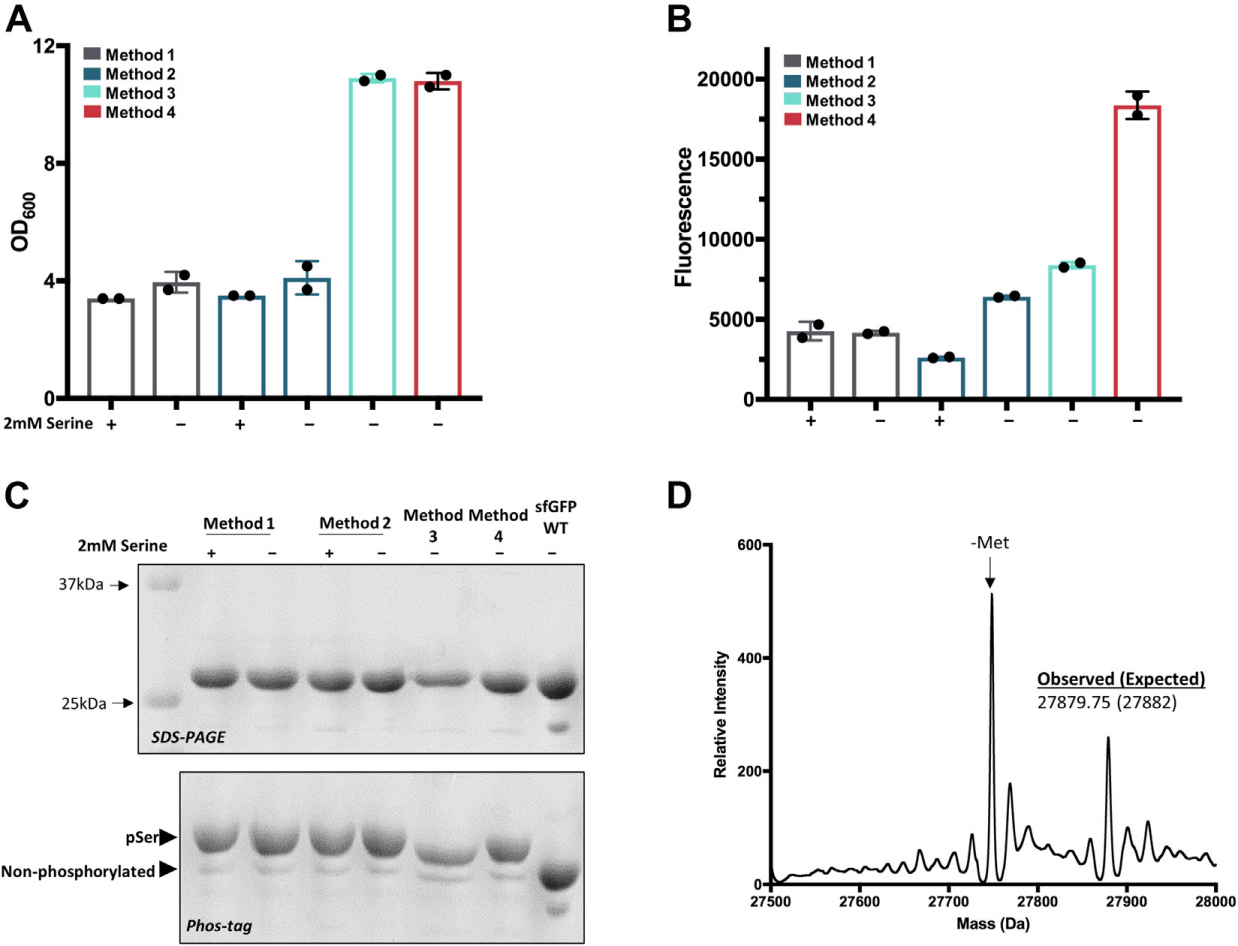
**Improving protein production with high-density culture protocols.** Final cell densities (panel *A*) and total culture fluorescence (panel *B*) of cultures expressing sfGFP-150pSer show that omission of free serine amino acid improved protein production for Method 2 (manual induction, low density). Expression at high-density further increased phospho-protein yield by ∼3-fold (Method 4). Error bars represent SDs of expressions performed in duplicate. *C*, purified sfGFP-150pSer proteins were >90% phosphorylated from all expressions (for full gels, see Fig. S1) and *D*, whole-protein mass spectrometry of sfGFP-150pSer produced with Method 4 further confirms homogenous phosphorylation. sfGFP, super folder GFP.

To this point, all sfGFP protein expressions were cultured in minimal media supplemented with 2 mM serine and 0.2% (w/v) Celtone, so we hypothesized that removing serine would improve protein yield without compromising protein phosphorylation by removing feedback inhibition of SerA (Fig. 1*A*) (32, 33, 34). For auto-induction expression (Method 1), we observed no notable defect in cell growth (Fig. 4*A*) nor increase in protein production when exogenous serine was omitted (Fig. 4*B*). On the other hand, with manual induction (Method 2), cell growth was similar (Fig. 4*A*) but phosphorylated protein yield increased 2-fold (Fig. 4*B*) compared to the same cultures containing serine and ∼30% higher than auto-induction expression (Method 1). In all cases, >90% of the purified protein was phosphorylated (Fig. 4*C*). Given the expense of isotopically labeled (^15^N and/or ^13^C) serine and that its omission does not have adverse side effects, serine was left out of subsequent expressions.

For these expressions, cells were grown to mid-log phase from an overnight non-inducing culture, at which time protein expression was induced and the final OD_600_ reached ∼3 to 5. To maximize protein yields per liter culture, we tested methods in which protein expression is performed at higher cell densities (Method 3 and 4 in Fig. 2) (37). We first tested a strategy in which cells were grown to stationary phase in ZY-NIM media overnight, resuspended in a Celtone supplemented minimal media, grown for a short period, and then induced manually at an OD_600_ ∼5 to 10 (Method 3 in Fig. 2). While the final OD_600_ at harvest was substantially higher than low-density expressions (Methods 1 and 2), total protein culture fluorescence was only modestly improved (Method 3, Fig. 4*B*), indicating protein production per cell was compromised, perhaps because the cells had not recovered from being in stationary phase when induction began. Alternatively, we inoculated fresh ZY-NIM media with the overnight culture, allowed it to grow to mid/late-log phase (OD_600_ ∼3–4) at which time the freshly grown cells were pelleted and resuspended in a Celtone supplemented minimal media and grown for a short period prior to IPTG induction (Method 4 in Fig. 2, adapted from (37)). The final cell density reached ∼8 to 12 like Method 3, however, overall protein production was >3-fold improved compared to the low-density cultures (Fig. 4*B*). The fluorescence values of these cultures correspond to approximately ∼8 to 10 mg of sfGFP-pSer150 per liter culture. Accurate incorporation of pSer was confirmed by Phos-tag gels and whole protein mass spectrometry (Fig. 4, *C* and *D*). Thus, we selected Method 4 for future isotopically labeled expressions.

#### Production of ^15^N sfGFP-pSer150 and ^1^H-^15^N HSQC spectra

**Figure 5:**
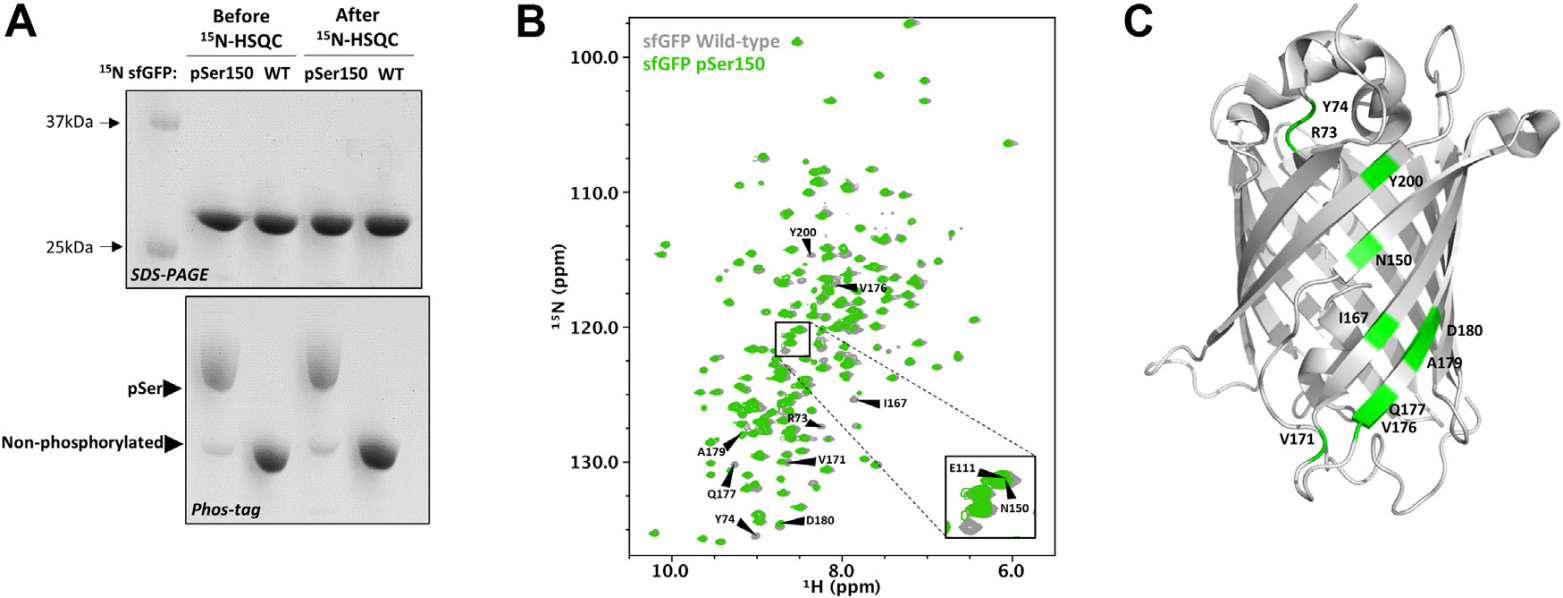
**Production and**^**1**^**H-**^**15**^**N HSQC analysis of**^**15**^**N-sfGFP proteins.***A*, SDS-PAGE and Phos-tag gel electrophoresis of purified ^15^N-sfGFP proteins before and after data collection demonstrate stability of phosphorylation even at 42 °C (for full gels, see Fig. S1) (38, 39). *B*, ^1^H-^15^N-HSQC spectra of wild-type sfGFP (*gray*) and 150pSer (*green*) in panel *A* were visualized and overlayed in NMRViewJ. Inset shows zoom in of peaks corresponding to E111 and N150. Resonances with chemical shift perturbation in 150pSer HSQC spectrum are labeled and *C*, highlighted on wild-type sfGFP 3D structure (highlighted *green*). sfGFP, super folder GFP.

Using Method 4, we produced ^15^N-labeled, >90% phosphorylated sfGFP at site N150, as well as WT sfGFP, to confirm uniform isotopic enrichment and subsequent utility for NMR analysis (Fig. 5*A*). Yields of purified sfGFP-150pSer were about half that of WT sfGFP (data not shown). Phos-tag gel electrophoresis confirmed >90% pSer incorporation before and after heteronuclear single quantum coherence (HSQC) data collection at 42 °C (Fig. 5*A*) (38, 39). Guided by previous backbone assignments of GFPuv and WT sfGFP (38, 39), we mapped the peak corresponding to residue 150 in the spectrum (8.5 ppm/119.8 ppm), which overlaps with residue E111 (Fig. 5*B*). Our sfGFP-150pSer transverse relaxation-optimized spectroscopy HSQC spectrum matches well with previously published WT sfGFP spectrum (38) with the exception of several perturbed resonances corresponding to residues near site 150.

We did not observe a clear down-field shift of the site 150 resonance in response to phosphorylation, however, which would be expected if a hydrogen bond was formed between the phosphate group and amide backbone (40). But being located within a β-sheet, the backbone amide of residue 150 is not expected to form such a hydrogen bond while also maintaining the native fold (41) (Fig. 5*C*). Despite lacking a clear assignment of the pSer150 cross peak, Phos-tag gel electrophoresis confirmed >90% phosphorylation both before and after HSQC data collection at 42 °C (Fig. 5*A*) (38, 39).

#### Phosphorylated SARS-CoV-2 Ser/Arg-rich linker region

As a biologically relevant example, we produced the Ser/Arg-rich IDR of the SARS-CoV-2 Nucleocapsid (N) protein isotopically labeled and with pSer at site S188. This region of N (residues 175–247) connects its RNA-binding domain and dimerization domain, and hyperphosphorylation of this SR-Linker region is thought to facilitate the release of viral gRNA from N (42, 43, 44). The mechanisms of this process are not well understood, though residues S188 and S206 within the SR-Linker of N when phosphorylated serve as “priming” sites for subsequent poly-phosphorylation by Glycogen Synthase Kinase-3β. Indeed, the N double mutant S188A/S206A renders the virus unable to replicate (13). Thus, the ability to produce site-specifically phosphorylated SR-Linker variants of N for NMR dynamics analysis could help elucidate this key step of SARS-CoV-2 life cycle.

**Figure 6:**
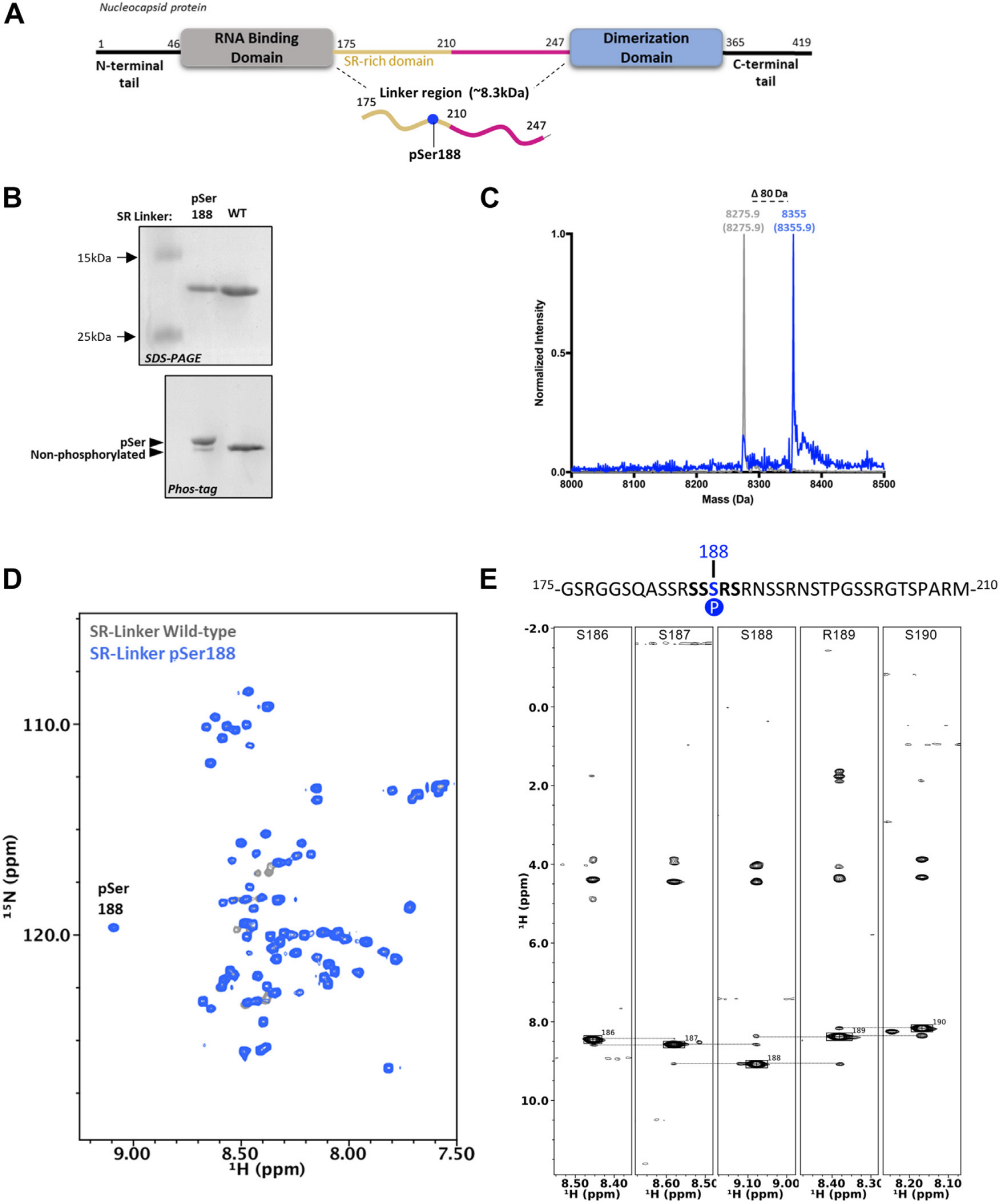
**Analysis of the SR Linker region of the N protein phosphorylated at S188.***A*, the linker region of SARS-CoV-2 N phosphoprotein contains a serine/arginine (SR) rich region that is phosphorylated in virions. This SR-Linker (175–247) is genetically fused to a TEV cleavable C-terminal sfGFP protein. *B*, purified unlabeled SR-Linker 188pSer are ∼80 to 90% phosphorylated as confirmed by 15% SDS-PAGE, Phos-tag gel electrophoresis (for full gels, see Fig. S1) and *C*, whole-protein mass spectrometry. *Dashed line* indicates +80 Da increments from wild-type SR-Linker (*gray*) to 188pSer (*blue*). *D*, ^1^H-^15^N HSQC spectra of wild-type SR-Linker (*gray*) and 188pSer (*blue*) is typical of an intrinsically disordered protein with pSer 188 chemical shift ^1^H-^15^N at 9.1 ppm and 119.6 ppm. *E*, amino acid sequence of the Ser/Arg-rich intrinsically disordered region of SARS-COV-2 with pSer188 highlighted in *blue* (*top*). The sequential assignment of backbone amide NOE’s for residues 186 to 190 visualized in SR-Linker pSer 188 3D-^15^N-NOESY-HSQC strip plot matches with amino acid sequence of SR-Linker (*bottom*). This confirms 9.1 ppm/119.6 ppm cross-peak observed in *D* belongs to pSer188. HSQC, heteronuclear single quantum coherence; NOE, nuclear overhauser effect; NOESY, nuclear overhauser effect spectroscopy; pSer, phosphoserine.

We expressed the SR-Linker region of N as a fusion with cleavable tags at its N- and C-terminus to improve solubility (*bd*SUMO and TEV-sfGFP-His6, respectively) first using Method 4 in unlabeled media. Purification and removal of the *bd*SUMO and sfGFP-His6 tags yielded pure SR-Linker with ∼80 to 90% phosphorylation at site S188 as confirmed by Phos-tag electrophoresis and whole-protein mass spectrometry (Fig. 6, *B* and *C*). Having confirmed production of SR-Linker in sufficient quality, we repeated the expression in an isotopically enriched culture medium. ^1^H-^15^N HSQC spectra at 10 °C of the purified proteins are characteristic of an IDR where signals cluster in the 8.0 to 8.5 ppm region due to poor dispersion in ^1^H spectra and matched well with previously published spectrum of a similar SR-Linker N construct (Fig. 6*D*) (45). In these spectra, a clear ^1^H-^15^N peak is observed at 9.1 ppm/119.6 ppm in the pSer188 protein sample that is not present in the WT sample, consistent with where a phosphorylated serine amide peak would be expected (Fig. 6*D*) (46, 47). To further confirm the assignment of the resonance at 9.1 ppm to pSer188, we collected a 3D-^15^N-nuclear overhauser effect spectroscopy (NOESY)-HSQC (Fig. 6*E*). We observed sequential backbone amide nuclear overhauser effects for residues ±2 from the pSer188 (Fig. 6*E*). These residues could be assigned based on the side chain proton chemical shifts, which were consistent with the expected amino acid sequence (Fig. 6*E*). Collectively, these data demonstrate the facile ability to generate sufficient quantities of a biologically relevant, phosphorylated IDR for NMR applications. Detailed structural and dynamics analyses of these proteins will be described in a subsequent article.

## Discussion

Here, we have described an optimized protocol for the generation of site-specifically phosphorylated, isotopically enriched proteins suitable for NMR analysis. The adoption of GCE pSer expression system imposed several challenges, including how to overcome growth deficiencies of the serine auxotroph BL21(DE3) Δ*serB* in minimal media, as well as enhanced phosphatase activity not typically seen when expressing phospho-proteins in standard rich media. Through rounds of optimization, we found three important parameters that facilitated homogenous phospho-protein production in labeling media: (i) Celtone as a key additive needed for healthy cell growth to overcome serine auxotrophy, (ii) Sep-tRNA_CUA_^v2^ for reduced mis-acylation by endogenous synthetases to minimize mis-incorporation of natural amino acids, and (iii) protein expression at lower temperatures to minimize phosphatase activities. Subsequent adaption of high-density methods allowed us to produce ≥10 mg purified sfGFP per liter culture with >90% phosphorylation, costing approximately ∼$300 per liter of uniformly ^15^N-labeled proteins. Although not demonstrated it here, this method can be adapted for ^13^C labeling as well by using ^13^C-labeled Celtone and glucose.

The ability to produce isotopically labeled phospho-proteins with GCE has been reported previously, but only in three instances to date. Two of these reports used the BL21(DE3) Δ*serB* strain but their methods did not describe a mechanism to overcome serine auxotrophy, and they also added unlabeled pSer amino acid to the culture media so that the expressed protein presumably has a mixture of labeled and unlabeled pSer (48, 49). In a third instance, recent work by Scheffner *et al.* (50) circumvented serine auxotrophy by using BL21(DE3) with intact SerB, and so the expression required the addition of high concentrations of unlabeled pSer to drive incorporation and downstream work benefited from the ability to purify away unphosphorylated populations by anion exchange chromatography, which may not always be possible. In the methodologies reported here, all pSer incorporated into the protein is biosynthesized from isotopically enriched media components, and thus, all pSer residues are expected to be isotopically labeled and will be visible in NMR spectra as demonstrated with ^15^N-HSQC data of phosphorylated SR Linker at site 188 (Fig. 6*D*).

The methods described here were optimized to express phosphorylated sfGFP and SR-Linker proteins, but we anticipate different proteins will require additional adjustments in expression protocols for specific applications. We found Celtone used at relatively low concentrations (0.2% (w/v)) to be an economically viable supplement to support phospho-protein expression; however, other commercially available isotopically labeled media additives such as BioExpress (Cambridge Isotope Laboratories, Inc), SILEX Media (Silantes), and ISOGRO (Sigma-Aldrich) may also be tractable. We believe that multi-site pSer incorporation should be possible, though feasibility will depend on the protein of interest and sites of incorporation. Adoption of other Δ*serB* strains of *E. coli*, such as the RF1-deficient B95(DE3) Δ*A* Δ*fabR* Δ*serB* (28), may prove helpful in this regard. However, our attempts to express sfGFP-150pSer in this strain using the methods reported here resulted in undesirable quantities of near-cognate suppression at the intended site of phosphorylation (data not shown), and so additional optimizations will be required for expressing isotopically labeled pSer proteins in this “truncation free” expression host. Nevertheless, the work here provides an important framework by which isotopically labeled, site-specific pSer-containing proteins can be expressed efficiently in *E. coli* and opens the door to more routine analyses of phosphorylated proteins with two-dimensional and three-dimensional NMR experiments.

## Experimental procedures

### Strains and plasmids

The BL21(DE3) Δ*serB* strain was a gift from Jesse Rinehart (Addgene #34929). BL21(DE3) and DH10b strains of *E. coli* were purchased from ThermoFisher Scientific. The pRBC-sfGFP WT and pRBC-sfGFP-150TAG plasmids were as previously described (Addgene #174075 and 174076, respectively) (28) (Fig. S2). The pKW2-EFSep was a generous gift from Jason Chin (Addgene # 173897) (Fig. S2). Genes for *bd*SUMO, *bd*SENP1, Sep-tRNA^v2^, and SARS-CoV-2 SR-Linker were synthesized by Integrated DNA Technologies. The SARS-CoV-2 SR-Linker protein (residues 175–24) expression plasmids were made by fusing a *bd*SUMO fusion protein (lacking the first 19 residues) at its N-terminus (51) and a TEV cleavable sfGFP-His_6_ at its C-terminus. All cloning steps were performed by SLiCE (52). The PPY strain used to generate SLiCE cloning extract was a gift from Yongwei Zhang (Albert Einstein College of Medicine) (52).

### Molecular biology reagents

Oligonucleotide primers were synthesized by Integrated DNA Technologies. Molecular biology reagents including restriction enzymes, T4 ligase, and polymerases were purchased either from Thermo Fisher Scientific or New England Biolabs. DNA Miniprep, Midiprep, PCR cleanup, and gel extraction kits were purchased from Machery Nagel. L-Serine, Celtone base powder (#1030P-U and 1030-N for unlabeled and ^15^N labeled, respectively), and 100X MEM Vitamin were purchased from Sigma-Aldrich, Cambridge Isotope Laboratories, Inc, and Thermo Fisher Scientific, respectively. Phos-tag Acrylamide for gel electrophoresis was purchased from NARD Institute, Ltd.

### Cell growth assessment

BL21(DE3) and BL21(DE3) Δ*serB* cells were streaked on LB/agar without antibiotics and grown overnight at 37 °C. A single colony was used to inoculate a buffered, glucose-rich ZY-NIM (Table 1) overnight with shaking at 250 rpm. The overnight starter was diluted to a starting optical density (OD_600_) of 0.15 into 50 ml of fresh minimal manual-inducing media (NIM-1) containing no additives, 2 mM serine, 0.2% Celtone, or both. Celtone was prepared by resuspending base powder with sterile water to a final concentration of 0.2% (w/v). Cells were grown for 24 h with OD_600_ measurements taken every hour for 9 h and once after 24 h. Cultures were grown in duplicate.

### Protein expression in minimal media

Fresh transformations were performed for all expressions in this study. BL21(DE3) Δ*serB* cells were cotransformed with either pKW2-EFSep containing either Sep-tRNA_CUA_(B4) or Sep-tRNA_CUA_^v2^ and the appropriate pRBC plasmid. Approximately, a dozen colonies were used to inoculate overnight ZY-NIM (Table 1) and grown at 37 °C. All ZY-NIM cultures contained 100 μg/ml ampicillin and 25 μg/ml chloramphenicol, while all minimal cultures contained 50 μg/ml ampicillin and 15 μg/ml chloramphenicol. Where indicated, L-serine was added at 2 mM final concentration and Celtone at 0.2% (w/v). To produce ^15^N-labeled proteins, NH_4_Cl was replaced with ^15^NH_4_Cl and Celtone replaced with ^15^N-Celtone.

#### Method 1: Minimal auto-induction media

Overnight ZY-NIM cells (OD_600_ ∼5–8) were pelleted by centrifugation at 5000 rcf and then resuspended into minimal AIM (Table 2) (starting OD_600_ ∼0.1–2). Cultures were grown at either 37 °C or 18 °C with shaking at 250 rpm in baffled flasks and harvested 24 or 48 h later, respectively.

#### Method 2: Minimal manual induction media, low density

Overnight ZY-NIM cells (OD_600_ ∼5–8) were pelleted by centrifugation at 5000 rcf and then resuspended minimal manual induction media 1 (MIM-1, Table 2) (starting OD_600_ ∼0.1–0.2). Cultures were grown at 37 °C until OD_600_ reached ∼0.6 to 0.8 and then induced with 1 mM IPTG. Cultures were grown at either 37 °C or 18 °C and harvested 24 or 48 h after IPTG addition, respectively.

#### Method 3: Minimal MIM, high density

ZY-NIM overnight starters (OD_600_ ∼5–8) were pelleted by centrifugation at 5000 rcf and resuspended into an equal volume of minimal MIM 2 (Table 2) (starting OD_600_ ∼5–8). Cultures were grown at 37 °C in baffled flasks until the OD_600_ increased by 1 to 2 units (∼1–2 h) and then induced with 1 mM IPTG. Cultures were grown at 18 °C for 48 h after IPTG addition.

#### Method 4: Minimal MIM, high density, with freshly grown cells

Cells from a ZY-NIM overnight starter culture (OD_600_ ∼5–8) were used to inoculate a fresh ZY-NIM culture (10% inoculum, *e.g.*, 5 ml into 50 ml fresh ZY-NIM). Cultures were grown at 37 °C with shaking at 250 rpm in baffled flasks until OD_600_ reached ∼3 to 4, at which point cells were pelleted by centrifugation at 5000 rcf and resuspended into an equal volume of minimal MIM 2 (Table 2) (starting OD_600_ ∼3–4). Cultures were grown at 37 °C in baffled flasks until the OD_600_ increased by 1 to 2 units (∼1–2 h) and then induced with 1 mM IPTG. Cultures were grown at 18 °C for 48 h after IPTG addition.

### Protein purification

#### sfGFP purification

Cell pellets containing sfGFP proteins were resuspended in Lysis Buffer (50 mM Tris pH 7.5, 500 mM NaCl, 5 mM imidazole, and phosphatase inhibitors: 50 mM sodium fluoride, 5 mM sodium pyrophosphate, 1 mM sodium orthovanadate) and lysed by microfluidization. Soluble cell lysate was obtained by centrifugation at 28,000 rcf for 45 min, to which TALON metal affinity resin was added. His_6_-tagged protein was allowed to bind to the TALON resin for 30 to 60 min with gentle rocking. Resin was collected and extensively washed with Lysis Buffer, and then protein was eluted with Lysis Buffer supplemented with 300 mM imidazole. After elution, proteins were further purified by gel-filtration on a 10/300 Superdex S75 column (Cytiva Life Sciences) in NMR buffer (30 mM sodium phosphate (pH 6.8), 100 mM NaCl) and then concentrated to 600 μM using a 10,000 Da cutoff filter prior to NMR analysis. SDS-PAGE and Phos-tag gels were poured immediately before use and run according to manufacturer recommendation.

#### SR-Linker purification

The SR-Linker of N was genetically fused to a *bd*SUMO cleavable N-terminal tag and a TEV cleavable C-terminal sfGFP protein for enhanced solubility (pRBC-bdSUMO-SR-Linker-sfGFP-His6). Cell pellets containing SR-Linker were resuspended in Lysis Buffer (50 mM Tris pH 7.5, 500 mM NaCl, 5 mM imidazole, and phosphatase inhibitors: 50 mM sodium fluoride, 5 mM sodium pyrophosphate, 1 mM sodium orthovanadate) and lysed by microfluidization. Soluble cell lysate was obtained by centrifugation at 28,000 rcf for 45 min, to which TALON metal affinity resin and 50 nM untagged *bd*SENP1 protease was added. The SR-sfGFP-His_6_ protein was allowed to bind to the TALON resin as the *bd*SENP1 protease cleaved the *bd*SUMO tag. Resin was collected and extensively washed with Lysis Buffer, and then SR-sfGFP-His_6_ was eluted with Lysis Buffer supplemented with and 300 mM imidazole. Purified protein was buffer exchanged into 50 mM Tris, 350 mM NaCl with phosphatase inhibitors using PD-10 desalting columns (Cytiva Life Sciences). The sfGFP-His_6_ tag was cleaved by TEV protease (1:20 TEV to SR) overnight at 4 °C, and the mixture was flowed through fresh TALON resin. The flow-through fraction contained SR-Linker protein while the TEV protease and sfGFP-His_6_ tag bound to the resin. For NMR analysis, SR-Linker protein was dialyzed overnight at 4 °C in 50 mM sodium phosphate, 150 mM NaCl, pH 6.5 buffer (without phosphatase inhibitors) and using a 3000 Da cutoff filter and concentrated to 100 μM prior to analysis.

### Quantification of sfGFP expression in cultures

Yield of sfGFP expressed per liter culture was calculated by measuring in-cell fluorescence of sfGFP and subtracting the contribution of cell auto-fluorescence (measured from the same density of cells not expressing any sfGFP construct). Fluorescence values were converted to mass of sfGFP per liter culture based on a standard curve of purified sfGFP. All values reported are the average of at least two independent replicate cultures, and error bars represent SDs.

### Whole protein mass spectrometry

Purified sfGFP proteins were exchanged into LC-MS grade water with PD-10 desalting columns. The SR-Linker proteins were buffer exchanged into 200 mM ammonium acetate by repeated concentration and dilutions using a 3000 Da cut-off centrifugal filter units. Mass spectra were obtained with a Waters Synapt G2 Mass Spectrometer at the Mass Spectrometry Facility at Oregon State University. The deconvoluted masses were obtained by using Waters MassLynx MaxEnt1 software.

### NMR analysis

NMR experiments were carried out on an 800-MHz Bruker Advance III HD NMR spectrometer equipped with a 5-mm triple resonance (HCN) cryogenic probe. Data collection for ^15^N-sfGFP proteins was carried out at 42 °C in 30 mm sodium phosphate, 100 mm NaCl (pH 6.8) buffer at a final concentration of 0.3 mM (38), while for ^15^N-SR Linker proteins, data were collected at 10 °C in 50 mM sodium phosphate, 150 mM NaCl, pH 6.5 buffer (53) at a final concentration of approximately 80 to 100 μM. All samples contained 10% D_2_O, 1 mM sodium azide, protease inhibitor mixture (Roche Applied Science), and 0.2 mm 2–2 dimethylsilapentane-5-sulfonic acid for ^1^H chemical shift referencing. All two-dimensional spectra were processed using NMRPipe (54) and visualized with NMRViewJ (55). To confirm the assignments of the pSer in the SR-Linker sample, we also collected a 3D-^15^N-NOESY-HSQC. The NOESY data were collected with a mixing time of 120 ms, 256 complex points in each indirect dimension, and 35% random nonuniform sampling. The three-dimensional data were processed using NMRPipe (54), SMILE (56), and visualized using NMRViewJ (55).

## Data availability

All data and reagents generated during this study are available from the corresponding author (rick.cooley@oregonstate.edu) upon request.

## Supporting information

This article contains supporting information (26, 28).

## Conflict of interest

The authors declare that they have no conflict of interest.
