# Supplementary material for: Accessing isotopically labeled proteins containing genetically encoded phosphoserine for NMR with optimized expression conditions
Source: J Biol Chem. 2022 Oct 17;298(12):102613. doi: 10.1016/j.jbc.2022.102613 (PMC9678770; doi:10.1016/j.jbc.2022.102613)
Supplement: Supplemental Figures S1 and S2 [file mmc1.docx]

**SUPPORTING INFORMATION**

**Accessing isotopically labeled proteins containing genetically encoded phosphoserine for NMR with optimized expression conditions**

Cat Hoang Vesely^1,2^, Patrick N Reardon^3^, Zhen Yu^2^, Elisar Barbar^2^, Ryan A. Mehl^1,2^, and Richard B. Cooley ^1,2*^

*From the* ^1^*GCE4 All Research Center*, ^2^*Department of Biochemistry and Biophysics, and* ^3^*Oregon State University NMR Facility, Oregon State University, Corvallis, Oregon, USA*

* To whom correspondence should be addressed: Oregon State University, Department of Biochemistry and Biophysics, 2011 Agricultural and Life Sciences, Corvallis, OR 97331

Telephone: (541) 737-4870 Fax: (541) 737-0481 Email: rick.cooley@oregonstate.edu

**Keywords:** post-translation modification; genetic code expansion; phosphoserine; nuclear magnetic resonance; protein synthesis


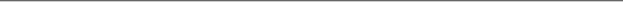


**
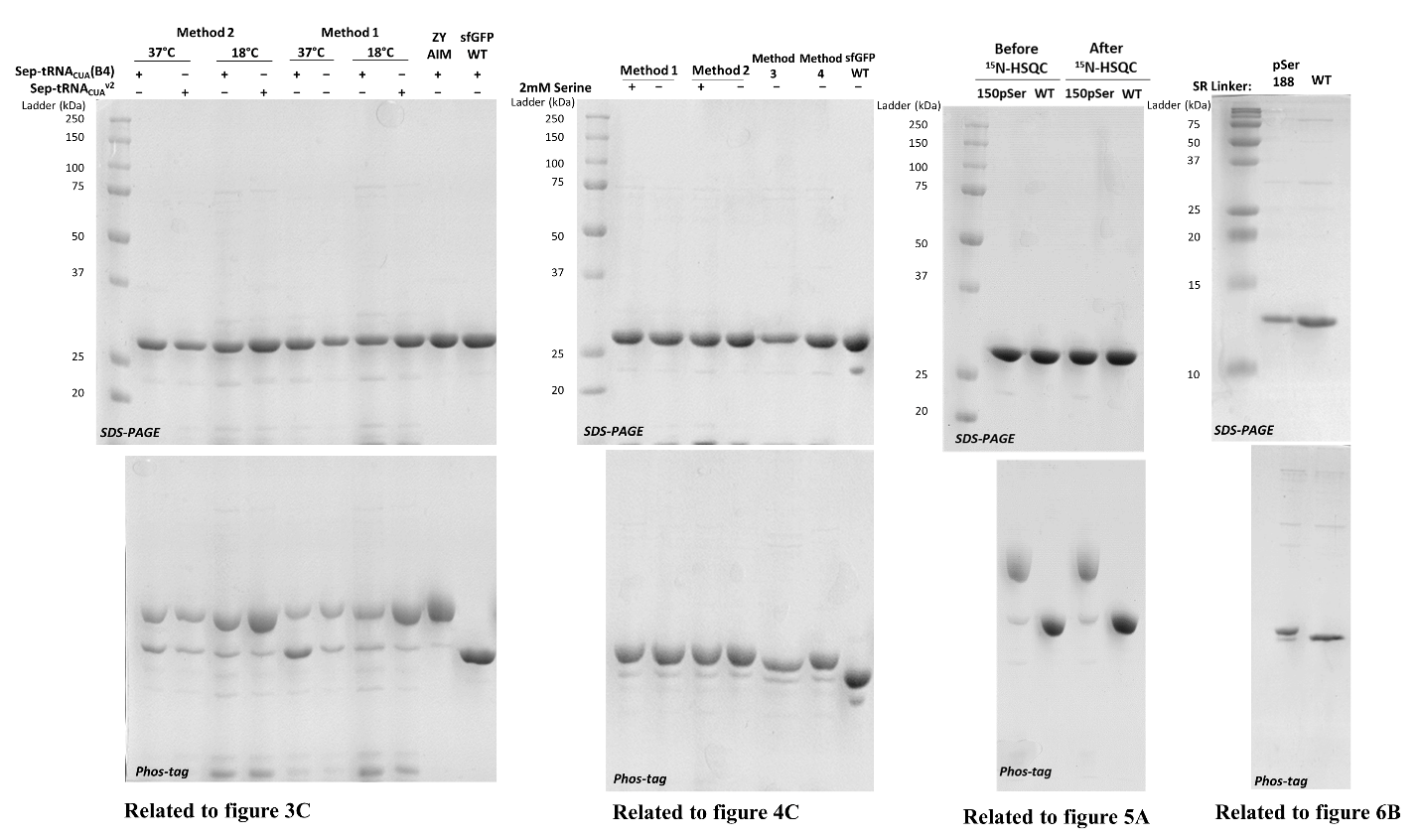
**

**Figure S1.** Full-length gel images corresponding to cropped images in this study.

**
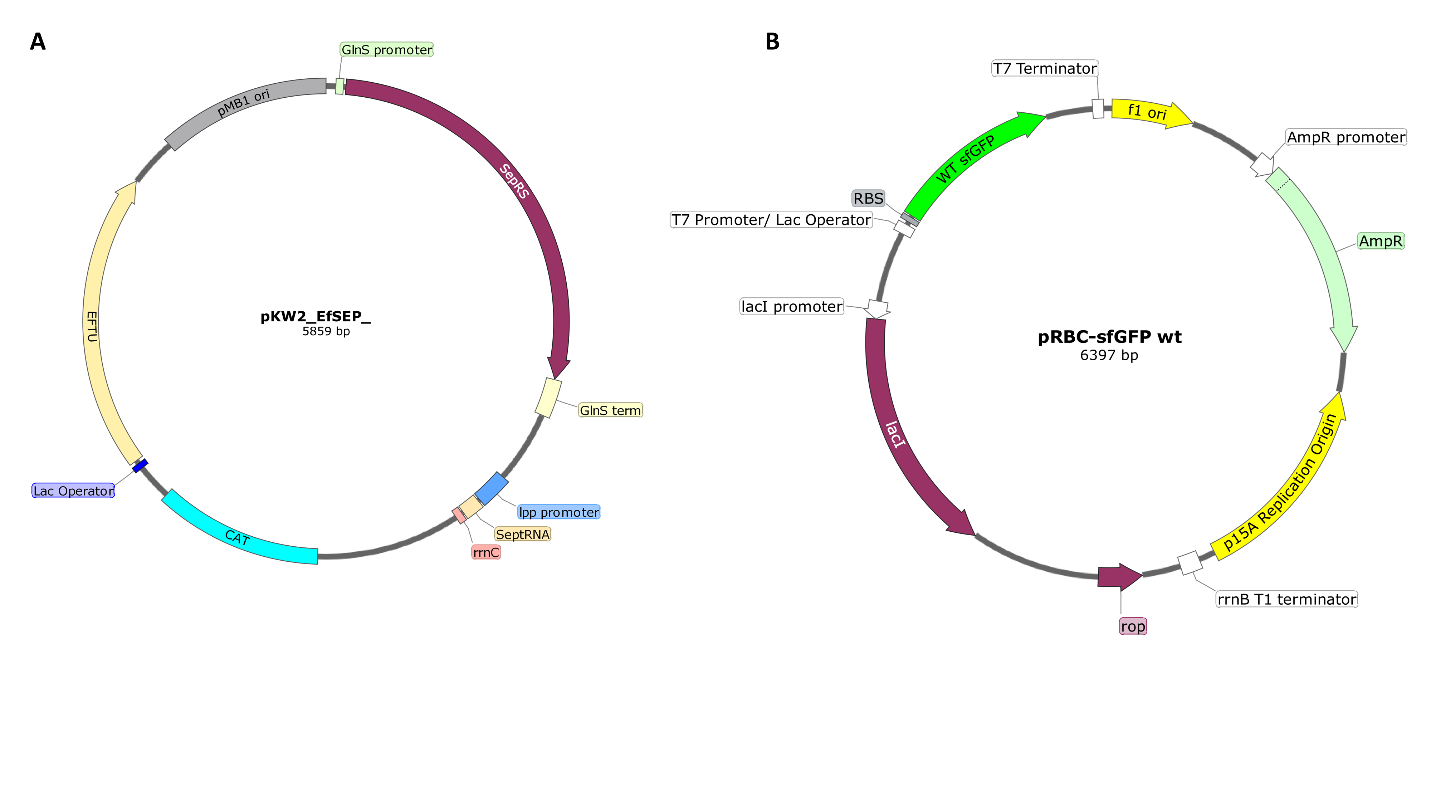
**

**Figure S2.** **Maps of plasmids used in this study.** *A,* pKW2_EFSep (1) and *B,* pRBC_sfGFP WT (2).

**References**

1. Rogerson, D. T., Sachdeva, A., Wang, K., Haq, T., Kazlauskaite, A., Hancock, S. M., Huguenin-Dezot, N., Muqit, M. M. K., Fry, A. M., Bayliss, R., and Chin, J. W. (2015) Efficient genetic encoding of phosphoserine and its nonhydrolyzable analog. *Nature Chemical Biology 2015 11:7*. **11**, 496–503

2. Zhu, P., Gafken, P. R., Mehl, R. A., and Cooley, R. B. (2019) A Highly Versatile Expression System for the Production of Multiply Phosphorylated Proteins. *ACS Chem Biol*. **14**, 1564–1572
